# Supplementary material for: Repeated COVID-19 vaccine boosters elicit variant-specific memory B cells in humans
Source: bioRxiv. 2025 Oct 20:2025.10.16.682893. Preprint. [Version 1] doi: 10.1101/2025.10.16.682893 (PMC12633353; doi:10.1101/2025.10.16.682893)
Supplement: 1 [file NIHPP2025.10.16.682893v1-supplement-1.pdf]

bioRxiv preprint doi: https://doi.org/10.1101/2025.10.16.682893; this version posted October 20, 2025. The copyright holder for this preprint (which was not certified by peer review) is the author/funder, who has granted bioRxiv a license to display the preprint in perpetuity. It is made available under aCC-BY 4.0 International license.

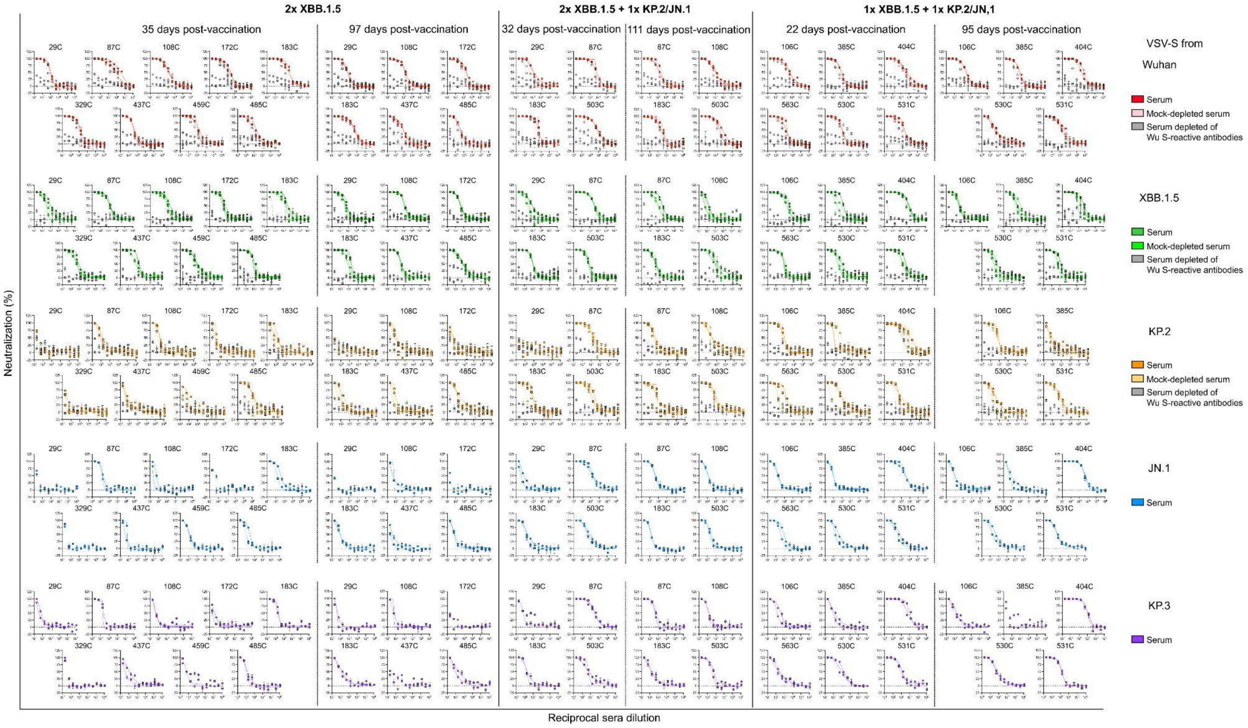

Figure S1, related to Figure 1 and 2. Serum neutralizing antibody titers after vaccination with XBB.1.5, JN.1 or KP.2 COVID-19 boosters. Dose-response curves against Wu/G614 S-, XBB.1.5 S, KP.2 S, JN.1 S and KP.3 S-VSV pseudoviruses mediated by human sera (Fig. 1) and by human sera that was

bioRxiv preprint doi: https://doi.org/10.1101/2025.10.16.682893; this version posted October 20, 2025. The copyright holder for this preprint (which was not certified by peer review) is the author/funder, who has granted bioRxiv a license to display the preprint in perpetuity. It is made available under aCC-BY 4.0 International license.

either mock-depleted or depleted of Wu S-reactive antibodies (Fig. 2). Data is presented as the average of technical duplicates with error bars representing the SEM from two biological experiments, squares symbols/dashed line or circles symbols/solid lines. Cohort member IDs are indicated on top of each graph.

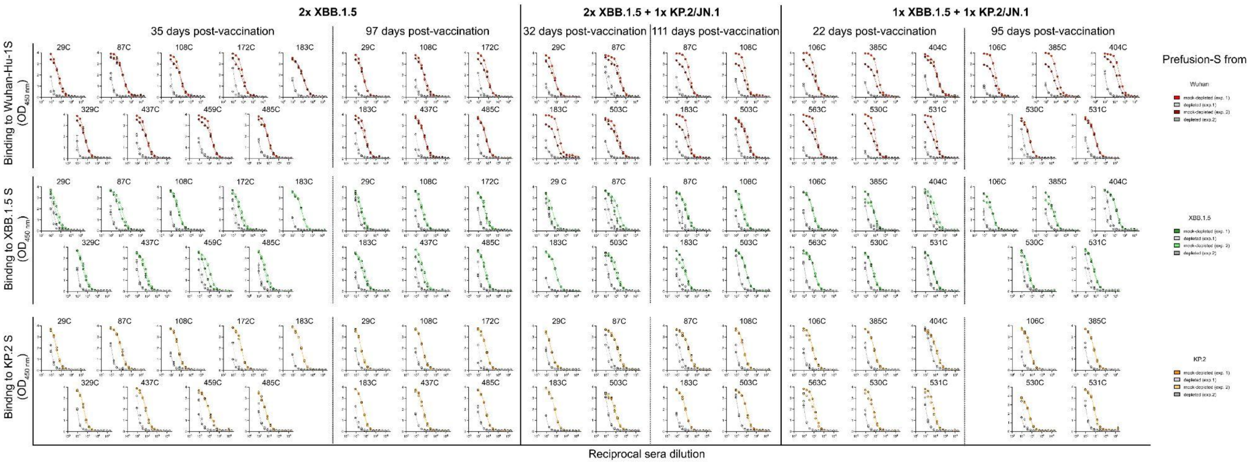

Figure S2, related to Figure 2. Serum antibody binding titers after vaccination with XBB.1.5, JN.1 or KP.2 COVID-19 boosters. Dose-response curves against prefusion Wu S, XBB.1.5 S and KP.2 S in human serum samples that were either mock-depleted or depleted of Wu S-reactive antibodies (grey). Data is presented as the average of technical duplicates with error bars representing the SEM from two biological experiments. Cohort member IDs are listed above each graph.

bioRxiv preprint doi: https://doi.org/10.1101/2025.10.16.682893; this version posted October 20, 2025. The copyright holder for this preprint (which was not certified by peer review) is the author/funder, who has granted bioRxiv a license to display the preprint in perpetuity. It is made available under aCC-BY 4.0 International license.

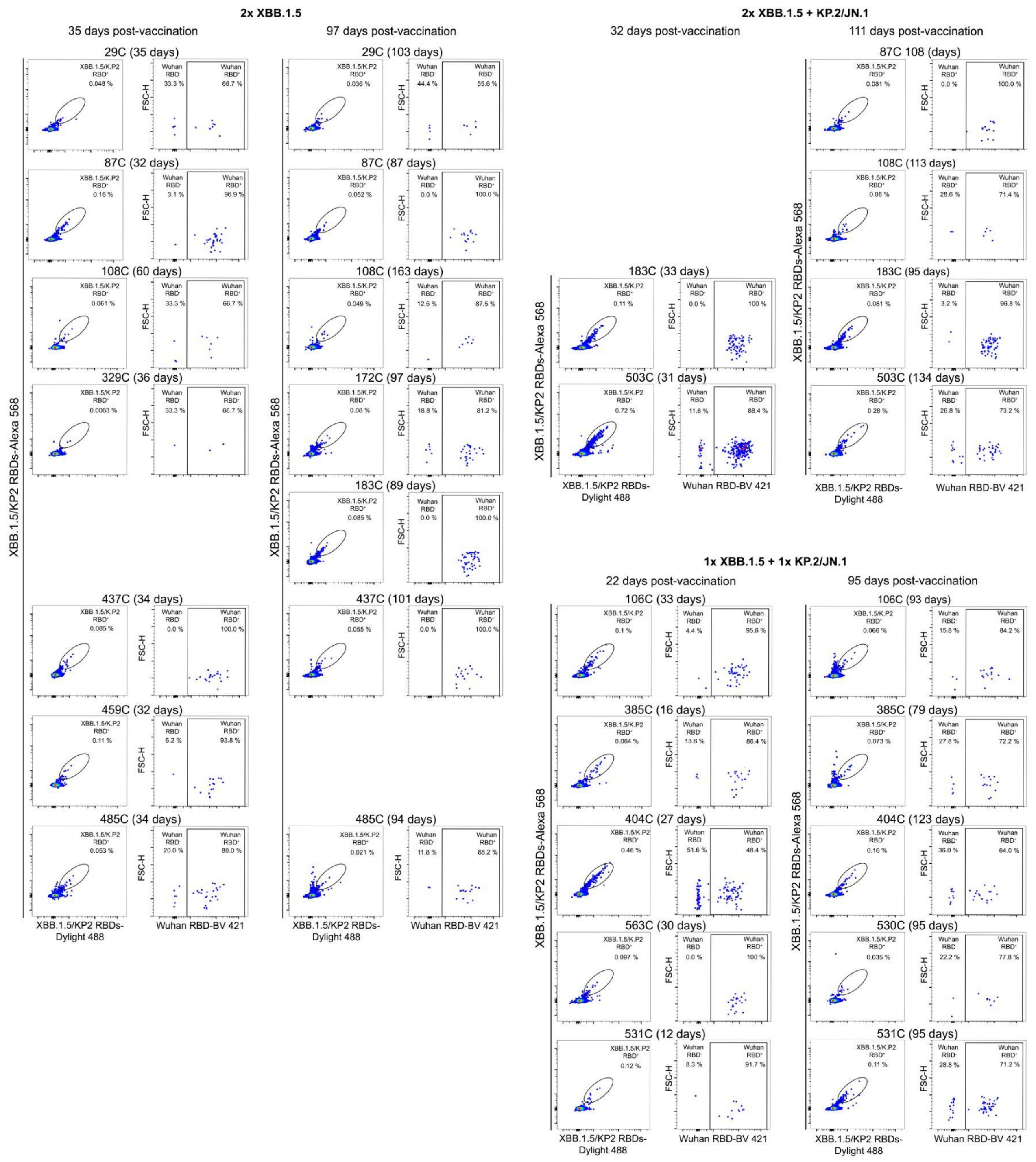

Figure S3, related to Figure 3. Flow cytometry analysis of memory B cells. Gating of XBB.1.5/KP.2 S RBD-reactive memory B cells and subsequent evaluation of Wu S RBD binding of these memory B cells from the peripheral blood of each individual collected at the indicated times from the 2x XBB.1.5 S (**A**), the 2x XBB.1.5 + 1x KP.2/JN.1 S (**B**) or 1x XBB.1.5 + 1x KP.2/JN.1 (**C**) cohorts using flow cytometry. The analysis was performed once.
